# Species Complex and Temporal Associations between Coccinellids and Aphids in Alfalfa Stands in Spain

**DOI:** 10.3390/insects12110971

**Published:** 2021-10-27

**Authors:** Roberto Meseguer, Alexandre Levi-Mourao, Xavier Pons

**Affiliations:** Agrotecnio-Cerca Center, Department of Crop and Forest Sciences, University of Lleida, Av. Alcalde Rovira Roure 191, 25198 Lleida, Spain; roberto.meseguer@udl.cat (R.M.); alexandrelevi.garcia@udl.cat (A.L.-M.)

**Keywords:** *Acyrthosiphon pisum*, *Therioaphis trifolii*, *Aphis craccivora*, *Coccinella septempunctata*, *Hippodamia variegata*, population dynamics, numerical responses

## Abstract

**Simple Summary:**

Alfalfa is the main fodder crop of the irrigated crop systems of northern Spain, as well as many Mediterranean countries. Alfalfa crops are damaged by some pest species but are also considered to be vast reservoirs of natural enemies of these pests. However, in Europe, the relationships between these pests and their natural enemies have been poorly studied. The aim of this study was to fill this gap in the knowledge. Therefore, we characterized the coccinellid species complex, identifying sixteen species, and the numerical relationships of the two that were most prevalent—*Coccinella septempunctata* and *Hippodamia variegata*—with the different aphid species, which are considered one of the alfalfa pests. The numerical relationships were separately studied in each of the alfalfa growing periods between cuttings (intercuts). Whereas the abundance of *C. septempunctata* was correlated with that of *A. pisum* in the second alfalfa intercut, *H. variegata* was mainly correlated with the abundance of *T. trifolii* in the fourth intercut as well as with the overall aphid abundance in the fifth intercut. This study helps to increase the knowledge on the predator–prey relationships, which is crucial for the proper pest management of alfalfa and the agricultural ecosystems in which it is included.

**Abstract:**

Alfalfa is known to be an important reservoir harboring natural enemies. The reduction in insecticide sprayings in recent years has allowed us to study the coccinellid species complex in this crop and the relationship between these predators and aphids. Alfalfa was sampled by sweep-netting throughout its productive period in several commercial stands each year between 2010 and 2021. The numbers and species of aphids and coccinellids were recorded. Sixteen coccinellid species were found. *Coccinella septempunctata* and *Hippodamia variegata* were, by far, the most prevalent species, with the former dominating during the first and second intercuts, whereas the latter dominated from the third to the fifth intercut. *Acyrthosiphon pisum* and *Therioaphis trifolii* were the most abundant aphid species, peaking in the second and fourth intercuts, respectively. Positive correlations were found between the abundance of *C. septempunctata* and *A. pisum* at the second intercut, between *H. variegata* and *T. trifolii* at the fourth intercut, and between *H. variegata* and the total number of aphids in the fifth intercut. This study helps to increase the knowledge on the predator–prey relationships of this crop and allows for designing strategies of conservation biological control against aphids.

## 1. Introduction

Alfalfa, *Medicago sativa* L., is the world’s most valuable cultivated forage crop [1]. In Spain, it is a traditional component of crop rotations, covering more than 253,000 ha in 2020 [2], which represents around 20% of the total surface used for this crop in Europe [3]. Spain is the main European exporter of alfalfa (dehydrated or in pellets), especially to Middle Eastern countries and China [4]. Stands usually remain in the field for 4 years and their management consists of regular cuttings during the growing season—usually, there are five that occur between the end of April and the end of September at intervals of 30–40 days.

Several pests can economically damage this crop—*Hypera postica* (Gyllenhal) (Coleoptera: Curculionidae), *Colaspidema barbarum* (Fabricius) (Coleoptera: Chrysomelidae), *Holotrichapion pisi* (Fabricius) (Coleoptera: Curculionidae), lepidopteran leaf feeders (several species), and aphids (Hemiptera: Aphididae).

The aphid species that occur in Spanish alfalfa stands are *Acyrthosiphon pisum* (Harris), *Aphis craccivora* Koch, and *Therioaphis trifolii* (Monell). Their seasonal occurrence, phenology, and population dynamics have been previously reported [5,6,7]. Besides the damage inflicted by their alimentary activity, they can transmit a wide range of viruses [8,9,10]. Although the number of insecticide treatments against aphids has been reduced over the last few years, some sprayings are still applied. In order to develop more sustainable control strategies, it is necessary to know which natural enemies are associated with alfalfa aphids in Spain, and their relationships. Among the most common ones are lady beetles (Coleoptera: Coccinellidae), pirate bugs (Hemiptera: Anthocoridae), damsel bugs (Hemiptera: Nabidae), hoverflies (Diptera: Syrphidae), and parasitoids (Hymenoptera: Braconidae, Aphidiinae) [6,11].

Studies on the occurrence and abundance of natural enemies of the alfalfa aphids in northern Catalonia have been conducted, where the relationships between aphids and parasitoids [7,11], and some heteropteran predators [6] were reported. Relationships between aphids and coccinellids were also investigated in the same area [6], with the results showing that significant numerical relationships only occurred during the growing alfalfa period between the first and the second cuttings. The fact that only two growing seasons were considered in the study and that the data came from a reduced area in northern Catalonia, close to the Pyrenees, could have mediated the results. Therefore, more information from a wider time period and crop cultivation area is needed in order to determine the coccinellid species complex, their numerical relationships with the main alfalfa aphid species, and their potential role as control agents.

The aims of the present work were to (1) characterize the coccinellid species complex in Spanish alfalfa stands, (2) determine their relative occurrence throughout the crop productive period, (3) define the aphid–coccinellid species associations, and (4) determine numerical and temporal relationships between the most abundant coccinellid and aphid species throughout the crop growing season.

## 2. Materials and Methods

**Figure 1:**
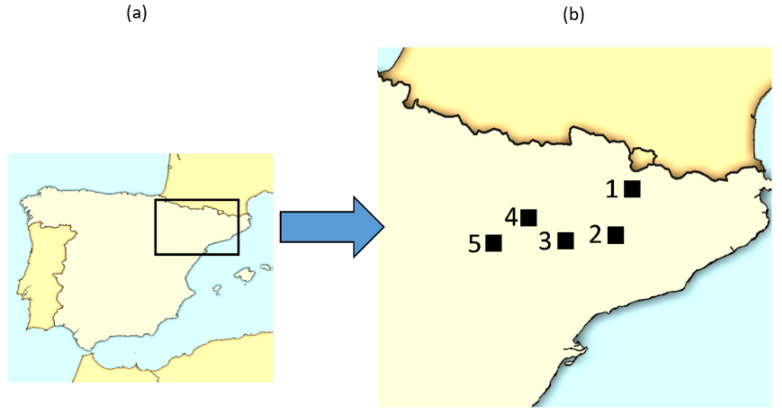
Zones showing the locations of the sampled fields. (**a**) Iberian Peninsula; (**b**) sampling areas in the northeast of Spain: (1) Alt Urgell (42°14′19″ N 1°24′22″ E); (2) Urgell (41°38′40″ N 0°54′46″ E); (3) Segrià (41°37′00″ N 0°37′00″ E); (4) Baja Cinca (41°54′00″ N 0°11′00″ E); and (5) Monegros (41°29′50″ N 0°09′13″ W).

The study was conducted along the Ebro Valley region, in the northeast of Spain, corresponding to 60% of the land surface used for alfalfa cultivation in Spain (and 20% considering Europe) (Figure 1).

A total of 112 alfalfa fields were sampled in the period from 2010 to 2021. All the sampled fields were sown with the Aragon variety, by far the most common one cultivated in the region [12]. Samplings were performed in each of the five growing alfalfa periods between cuttings (intercuts hereafter; see [6,11], from March to September). However, only 99 fields that did not receive insecticide sprayings were included in this study. As the study was part of a more extensive project dealing with the integrated pest management of alfalfa in the region, unequal numbers of fields were sampled in each of those intercuts, and more fields were sampled in the first one, when the most damaging pest *H. postica* occurred [13]. Each field was divided into four sectors, and three samples per sector were collected following the central part of one of the main diagonals and at least 25 m apart (following [6]). Samples were taken with a 38 cm diameter sweep net, sweeping it from side to side five times in a 180° arc. The samples were placed in an icebox and transported to the laboratory, where they were frozen and stored until aphid and coccinellid individuals were identified to the species level, following [14,15], and counted. For each aphid species, all morphs and instars were considered together, whereas the larvae and adults of the coccinellids were distinguished. One sample per intercut was obtained.

### Data Analysis

The total number of coccinellids of each species (adults and larvae) and aphids (all stages together) recorded in each sampling point of one field were averaged, and one field was considered as one replicate. Alfalfa cuttings involve a temporary but drastic change to the system, therefore the five intercuts were considered as separate units. As unequal numbers of fields were sampled in each intercut, the overall relative abundances of the different coccinellid species for the total study period were calculated based on standardized values (the number of each coccinellid species in each intercut was divided by the total number of sampled fields in that intercut).

Statistical analyses were performed with R version 4.0.3 (R Foundation for Statistical Computing, Vienna, Austria). The numerical relationships between coccinellid and aphid species in each intercut were determined by correlation analysis (cor function). Additionally, because we had data on the different *H. postica* larval instar abundances in the first intercut for some years and it has been reported as alternative prey for *Coccinella septempunctata* L. [16,17], the numerical relationship between these two species was also analyzed. The shapiro.test function was used to check for normality before and after transforming the data as (log (x + 1)). The data did not follow a normal distribution; therefore the Spearman correlation test was used. No correlation analyses were performed for the third intercut, as the aphid abundances were very low.

## 3. Results

### 3.1. Coccinellid Species Complex and Relative Abundance

A total of 9124 coccinellids were collected throughout the study. Sixteen species/genus were recorded: *C. septempunctata*, *Hippodamia variegata* (Goeze), *Propylea quatuordecempunctata* L., *Scymnus* spp., *Coccinella quinquepunctata* L., *Coccinula quatuordecimpustulata* L., *Exochomus nigromaculatus* (Goeze), *Tytthaspis sedecimpunctata* L., *Hyperaspis* sp., *Adalia bipunctata* L., *Oenopia lyncea lyncea* Olivier, *Coccinella undecimpunctata* L., *Subcoccinella vigintiquattuorpunctata* L., *Chilochorus bipustulatus* L., *Psyllobora vigintiduopunctata* L., and *Stethorus punctillum* (Weise). Whereas *C. septempunctata*, *H. variegata*, *P*. *quatuordecempunctata*, *C. quatuordecimpustulata*, *Scymnus* sp., *E. nigromaculatus, C. quinquepunctata*, *A. bipunctata*, *O. lyncea lyncea,* and *C. undecimpunctata* are aphidophagous predators, *Hyperaspis* sp. and *C. bipustulatus* are mainly coccidophagous, but consume aphids as secondary prey. *Tytthaspis sedecimpunctata* and *P. vigintiduopunctata* feed on fungus, especially powdery mildew. *Subcoccinella vigintiquattuorpunctata* is herbivorous and *Stethorus punctillum* is a mite predator.

The most abundant species were *C. septempunctata* and *H. variegata* (Table 1), both accounting for nearly 95% of species in the whole study. *Coccinella septempunctata* was the predominant species in the first and the second intercuts, making up around 80% of the collected coccinellids (Table 1). Although it was present throughout the entire alfalfa growing season, its relative abundance decreased drastically from the third to the fifth intercut. The opposite trend was recorded for *H. variegata*, which was also present during the entire growing season, but it became the predominant species from the third to the fifth intercut, when it accounted for 76, 89, and 89% of the collected coccinellids, respectively (Table 1).

**Figure 2:**
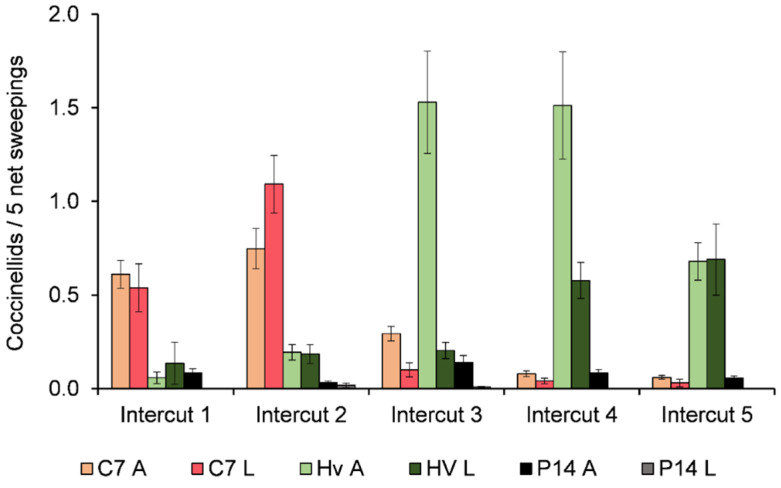
The mean number ± s.e. of adults (A) and larvae (L) per five net sweepings of the three main coccinellid species, *Coccinella septempunctata* (C7), *Hippodamia variegata* (Hv), and *Propylea quatuordecimpunctata* (P14) in each of the five intercuts of the growing season.

Regarding the population structure of *C. septempunctata* (Figure 2), 53% of the collected individuals in the first intercut were adults and the other 47% were larvae. The proportion of larvae increased to 60% in the second intercut. Later in the season, adults occurred more commonly than larvae.

Despite the low numbers, the population structure of *H. variegata* in the first intercut was mainly composed of larvae (70% larvae vs. 30% adults), but proportions of both development stages were equal in the second intercut (Figure 2). In the third intercut, many more adults were recorded, accounting for nearly 90% of the collected *H. variegata* individuals. This proportion steadily decreased to 72% and 50% in the fourth and fifth intercuts, respectively.

*Propylea quatuordecimpunctata* and *Scymnus* sp. were also common species, but only accounted for 4% and 1% of the total number of collected coccinellids, respectively (Table 1). Most of the individuals collected were adults. The occurrences of the other species were sporadic.

### 3.2. Aphid Abundance

**Figure 3:**
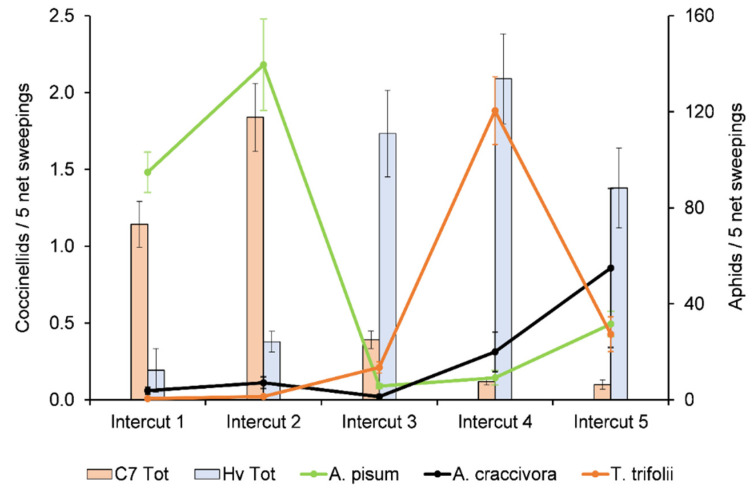
The mean abundance ± s.e. of the alfalfa aphids *Acyrthosiphon pisum*, *Therioaphis trifolii*, and *Aphis craccivora* in each of the five intercuts of the growing season, and the mean number ± s.e. of individuals (adults and larvae) per five net sweepings of the two main coccinellid species (bars), *Coccinella septempunctata* (C7) and *Hippodamia variegata* (Hv).

The occurrences of the three previously reported alfalfa aphid species in Spain—*A. pisum*, *T. trifolii*, and *A. craccivora*—were recorded. The former occurred during the whole growing season, but with remarkable differences between the intercuts (Figure 3). This species was prevalent in the first and second intercuts, peaking in the latter. A huge decrease in its population was recorded in the third and fourth intercuts, but it recovered later, at the end of the growing season. An opposite trend in population dynamics occurred with *T. trifolii* (Figure 3), where this species was either not collected or only sporadically collected in the first and second intercuts. A slight increase in its population was recorded in the third intercut, reaching its peak in the fourth. Then, a sharp decrease occurred in the fifth intercut. Despite the values shown in Figure 3, the occurrence of *A. craccivora* was very erratic, with some fields showing high populations whereas, in some others, populations were completely irrelevant. There was an increase in its abundance in the fifth intercut.

### 3.3. Numerical Relationships between Aphid and Coccinellid Species

**Figure 4:**
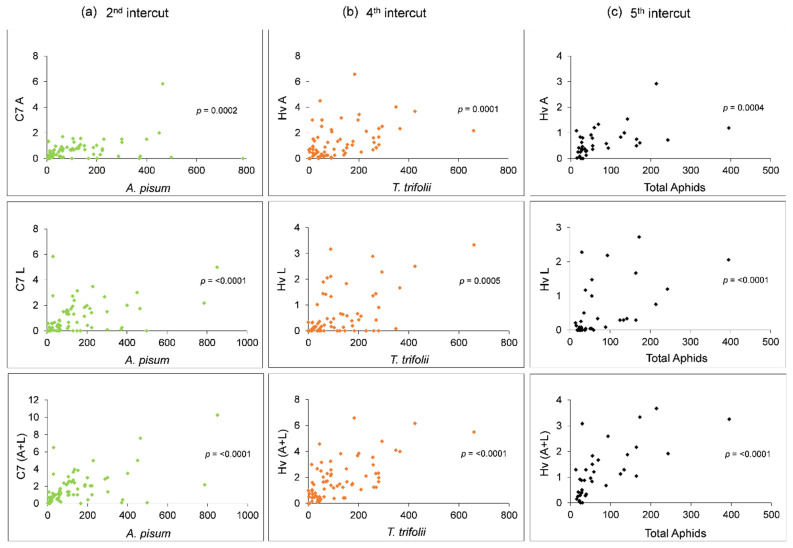
The most representative correlations: (**a**) *A. pisum–C. septempunctata* (second intercut); (**b**) *T. trifolii–H. variegata* (fourth intercut); and (**c**) total aphids–*H. variegata* (fifth intercut). C7, *C. septempunctata*; Hv, *H. variegata*; A, adults; and L, larvae.

Based on the relative abundance of the coccinellid species, we restricted the results to *C. septempunctata* and *H. variegata*. Looking at Figure 3, there seems to be a close relationship between the occurrence of *C. septempunctata* and *A. pisum* in the first two intercuts. However, no significant correlations between *C. septempunctata* adults, larvae, or both together with *A. pisum* were found in the first intercut (Table 2). Conversely, these variables were significantly and positively correlated in the second intercut (Figure 4a, Table 2). A close relationship between *H. variegata* and *T. trifolii* was also shown in the fourth intercut (Figure 3), which was confirmed by the correlation analyses, where *H. variegata* adults, larvae, and both together showed positive correlations with this aphid, the prevalent species at this time (Figure 4b, Table 2). *Hippodamia variegata* larvae were also positively correlated with *A. pisum* and *A. craccivora*. Positive correlations with the total number of aphids were also recorded for this coccinellid species (Table 2). However, these results are likely mediated by the abovementioned positive correlation with *T. trifolii*, the most abundant aphid in this intercut. Regarding the fifth intercut, *H. variegata* was well correlated with *A. pisum*, *A. craccivora*, and the total number of aphids, but not with *T. trifolii* (Figure 4c, Table 2).

### 3.4. Numerical Relationships between H. postica larvae and C. septempunctata

In the first intercut, no significant correlations were observed (*p* > 0.10) when we evaluated the numerical relationships between *C. septempunctata* and its potential alternative prey *H. postica*. However, after adding the values of the *A. pisum* and *H. postica* larvae abundances, the correlations improved, but statistically significant results were only obtained for *C. septempunctata* adults and *H. postica* L4 plus *A. pisum* (Table 3).

## 4. Discussion

Alfalfa crops are known to be significant reservoirs of natural enemies [5,11]. However, studies dealing with the predator/parasitoid–prey relationships in this crop are scarce in Europe. Increasing our knowledge in this area is crucial for the proper pest management of alfalfa and the agricultural ecosystems in which it is included. In this eleven-year-long study, new information on the coccinellid species complex and their relationships with the alfalfa aphids was revealed.

Results on the aphid complex species and their temporal occurrence did not differ from those previously reported in the study area [6]. However, our results show a change in the seasonal prevalence, as *A. pisum* was the species that reached the highest abundance values instead of *T. trifolii*, as reported in Pons et al. [6]. The current study considered a wider area that includes regions with milder temperatures during the first and second intercuts, which may have been favorable for *A. pisum* populations.

Sixteen different coccinellid species were recorded. Within them, *C. septempunctata* and *H. variegata* were the most prevalent. These results concur with those of other studies, which also reported these two species as being the dominant coccinellids in alfalfa stands in Europe [5,18]. Although the presence of the harlequin lady beetle, *Harmonia axyridis* Pallas, has been recorded in northeastern Spain [19], and this invasive coccinellid is usually found in urban green areas of the region (authors, unpublished), it was not recorded in this study. Following its invasion in Chile, this coccinellid species rapidly increased in abundance and became dominant in alfalfa stands [20]. If *H. axyridis* is able to colonize alfalfa stands in the study area, the relationships of native coccinellids with aphids will probably change [21].

Regarding the seasonal occurrence of *C. septempunctata* and *H. variegata*, our results show that both species share the same habitat. Contrary to what was reported in other studies [22], there was a clear succession in their prevalence; *C. septempunctata* dominated during the first and second intercut, while *H. variegata* dominated in subsequent intercuts. Three factors may have led to this succession, including the species phenology (crop colonization and the timing of departure), predator–prey species associations, and intraguild predation events. Assuming that both species overwinter as adults outside of alfalfa stands, it seems clear that *C. septempunctata* colonizes alfalfa earlier than *H. variegata*. However, reproduction may occur during the first intercut, and larvae were recorded for both coccinellid species. The non-significant correlation between *C. septempunctata* and *A. pisum*, practically the only aphid species present in the first intercut, suggests that aphids are probably not the only reason for the predominance of *C. septempunctata*. Aggregative and numerical coccinellid responses to aphids can be influenced by several factors [23,24], such as the presence of alternative prey [25]. We found a significant positive correlation between the number of *C. septempunctata* adults and that obtained after the addition of *A. pisum* and *H. postica* fourth instar larvae abundances, suggesting that the weevil can contribute to the reproduction of this species and, thus, to the development of *C. septempunctata* populations, as reported by Richards and Evans [17].

In the second intercut, the abundance of *C. septempunctata* was correlated with that of *A. pisum*, whereas *H. variegata* was not. Although this aphid species was relatively abundant in the first and second intercuts, the competence for prey resources and the intraguild predation events between these two coccinellid species could have contributed to this. The dominance of *C. septempunctata* over other coccinellid species has been reported in several studies [26,27]. In addition, intraguild predation between *C. septempunctata* and *H. variegata* has been already reported [28,29], and was asymmetric for the former. Such interactions could have postponed the proper establishment of *H. variegata* in alfalfa until the departure of *C. septempunctata*.

After the peak of *A. pisum* in the second intercut, its occurrence decreased drastically in the third intercut, as did the occurrence of *C. septempunctata*. This was likely due to the lack of prey leading to the adults leaving the alfalfa stands. Ricci et al. [30] reported that when aphids are scarce, coccinellid adults leave crops in search of new aphid food, which can be a plausible justification for this drop. This is reinforced by Madeira et al. [31] and di Lascio et al. [32], who observed that other common crops in northeastern Spain, such as maize, can have a “sink” effect during its vegetative growing period (coinciding with the third alfalfa intercut) over the alfalfa *C. septempunctata* populations, which do not return after leaving. Summer diapause has also been described as a common trait of Mediterranean *C. septempunctata* populations [33], so it should also be considered as a possible cause for this decrease. It could also be presumed that most of the larvae can be destroyed with the alfalfa cutting, but the records of Ghahramani et al. [22] seem to not corroborate this. However, if the cutting is advanced enough to control the large amount of damage caused by *C. barbarum*, a significant pest that affects the second intercut, larvae of *C. septempunctata* may not be able to reach adulthood and migrate from alfalfa, becoming seriously affected. More studies are needed to elucidate the phenology of this species in the Ebro Valley area.

The highest abundances of *H. variegata* adults were shown during the third and fourth intercuts. These results suggest that significant immigration to the alfalfa stands from the surrounding crops may occur during this period of the year. This massive entry could be related to the growing populations of *T. trifolii*, which were reported to act as an attracting and arrestant stimulus for *H. variegata* [34]. Di Lascio et al. [32] already reported the movement of *H. variegata* individuals between alfalfa and maize crops in this period. The better adaptation for the reproduction of this coccinellid species during the summer [35,36] is probably the cause of its dominance from the third to the fifth intercuts.

The positive correlations recorded for *H. variegata* in the fourth and fifth intercuts partially differ from the results of Pons et al. [6], who only reported a positive correlation between this coccinellid species and *A. craccivora* in the fifth intercut. As mentioned above, the abundant presence of *T. trifolii* during the fourth intercut may have acted as an attracting and arrestant stimulus for *H. variegata* individuals and, thus, may have led to their positive correlation. During the fifth intercut, the dominance of *A. craccivora*, which was already described as very suitable prey for *H. variegata* [37], may explain its positive correlation with this aphid species and, thus, with the total number of aphids. The sharp decrease in *T. trifolli* abundance recorded in this intercut could be due to its own phenology, but the predation of *H. variegata* and, possibly, other specific natural enemies as parasitoids may also have contributed. *Trioxys complanatus* Quilis and *Praon exsoletum* (Nees) have been reported as the parasitoid species associated with *T. trifolii* in the northern area of the study region [11]. The rate of parasitism by these two species was estimated between 5% and 15%, a low to moderate rate compared with the rates of other alfalfa aphids [7]. An extensive study on the aphid parasitism is being carried out in the whole Ebro Valley in order to better know the effective role of parasitoids in alfalfa aphid control.

The abundance of *P. quatuordecimpunctata*, even as the third most frequently occurring species, was very low when compared to *C. septempunctata* and *H. variegata*. The numbers of larvae collected were especially low, and this suggests that adults of this species did not significantly reproduce in alfalfa stands. On the other hand, no correlation with aphids was found (data not shown).

Our study presents previously unreported, positive coccinellid–aphid correlations, such as between *H. variegata* and *T. trifolii*, as well as new information about the coccinellid complex of alfalfa in the Ebro Valley region. More studies are needed in order to increase the knowledge on the predator–prey interactions of this crop in Europe.

## 5. Conclusions

Based on this eleven-year-long study, we present the first report on the coccinellid species complex in Spanish alfalfa stands and the relationships with aphids. Sixteen coccinellid species were recorded, among which eight were aphidophagous. *Coccinella septempunctata* and *H. variegata* were the prevalent species, but a clear succession between them was observed; the former dominated during the first and second intercuts, whereas the latter dominated from the third to the fifth intercut. Several positive correlations were found, but the most significant were those between the abundance of *C. septempunctata* and *A. pisum* in the second intercut, between *H. variegata* and *T. trifolii* in the fourth intercut, and between *H. variegata* and the total number of aphids in the fifth intercut. This study contributes to increase the knowledge on the predator–prey relationships of this crop, knowledge that remains scarce in Europe.

## Figures and Tables

**Table 1:** The numbers of individuals collected and relative abundance (% in parenthesis) of the different coccinellid species per intercut, and the standardized relative abundance (%) for the total sampled period. *C7*: *Coccinella septempunctata*; *Hv*: *Hippodamia variegata*; *P14*: *Propylea quatuordecimpunctata*; *Scy*: *Scymnus* sp.; and *Stet*: *Stethorus punctillum*.

No. Fields	Intercut	*C7*	*Hv*	*P14*	*Scy*	Other Aphidophagous	*Stet*	Others	TOTAL
99	1	1696 (82.57)	230 (11.20)	102 (4.97)	18 (0.88)	3 (0.15)	1 (0.05)	4 (0.19)	2054 (100.00)
77	2	1700 (79.81)	351 (16.48)	47 (2.21)	19 (0.89)	0 (0.00)	12 (0.56)	1 (0.05)	2130 (100.00)
70	3	329 (15.30)	1629 (75.77)	128 (5.95)	44 (2.05)	8 (0.37)	1 (0.05)	11 (0.51)	2150 (100.00)
75	4	108 (5.14)	1882 (89.49)	73 (3.47)	20 (0.95)	3 (0.14)	13 (0.62)	4 (0.19)	2103 (100.00)
37	5	44 (6.40)	611 (88.94)	26 (3.78)	4 (0.58)	1 (0.15)	1 (0.15)	0 (0.00)	687 (100.00)
**Standardized relative abundance (%)**									
		37.01	57.07	4.09	1.14	0.17	0.30	0.21	100

**Table 2:** Spearman’s correlation coefficients (rho) and *p*-values between the most abundant aphid species and coccinellids in four of the five intercuts of the alfalfa growing season (the third intercut was not included due to the low abundance of aphids). C7, *C. septempunctata*; Hv, *H. variegata*; A = adults; L = larvae; and -- = no correlation analyses were performed.

	*A. pisum*		*T. trifolii*		*A. craccivora*		Total Aphids	
	rho	*p*-Value	rho	*p*-Value	rho	*p*-Value	rho	*p*-Value
1st intercut								
C7 (L + A)	0.0041	0.9686	--	--	--	--	--	--
C7 A	0.0484	0.6433	--	--	--	--	--	--
C7 L	−0.0059	0.9548	--	--	--	--	--	--
Hv (L + A)	0.1309	0.2085	--	--	--	--	--	--
Hv A	0.0624	0.5502	--	--	--	--	--	--
Hv L	0.1834	0.0761	--	--	--	--	--	--
2nd intercut								
C7 (L + A)	0.5651	<0.0001	--	--	--	--	--	--
C7 A	0.4202	0.0002	--	--	--	--	--	--
C7 L	0.5211	<0.0001	--	--	--	--	--	--
Hv (L + A)	0.2522	0.0325	--	--	--	--	--	--
Hv A	0.1968	0.0976	--	--	--	--	--	--
Hv L	0.0772	0.5189	--	--	--	--	--	--
4th intercut								
Hv (L + A)	0.0320	0.7936	0.5880	<0.0001	0.3417	0.0078	0.6012	<0.0001
Hv A	−0.2490	0.0391	0.4507	0.0001	−0.0576	0.6382	0.4088	0.0005
Hv L	0.5295	<0.0001	0.4103	0.0005	0.5731	<0.0001	0.4352	0.0002
5th intercut								
Hv (L + A)	0.4433	0.0068	0.1900	0.2671	0.7298	<0.0001	0.7248	<0.0001
Hv A	0.3395	0.0427	0.3818	0.0216	0.4780	0.0040	0.5541	0.0004
Hv L	0.4398	0.0073	0.0111	0.9460	0.6518	<0.0001	0.6310	<0.0001

**Table 3:** Spearman’s correlation coefficients (rho) and *p*-values between the different larval instars of *H. postica* and the lady beetle *C. septempunctata* in the first intercut. C7, *C. septempunctata*; A, adults; L, larvae; L1, first instar; L2, second instar; L3, third instar; and L4, fourth instar.

	*H. postica* L1-L2 + *A. pisum*		*H. postica* L3 + *A. pisum*		*H. postica* L4 + *A. pisum*		*H. postica* Total + *A. pisum*	
	rho	*p*-Value	rho	*p*-Value	rho	*p*-Value	rho	*p*-Value
C7 (L + A)	0.2408	0.0553	0.2113	0.0936	0.2108	0.0945	0.1982	0.1164
C7 A	0.2005	0.1121	0.2152	0.0876	0.2526	0.0440	0.2046	0.1049
C7 L	0.1351	0.2648	0.0933	0.4422	0.0958	0.4296	0.0788	0.5165

## Data Availability

The data presented in this study are available on request from the corresponding author.
